# Transparent PAN:TiO_2_ and PAN-co-PMA:TiO_2_ Nanofiber Composite Membranes with High Efficiency in Particulate Matter Pollutants Filtration

**DOI:** 10.1186/s11671-019-3225-2

**Published:** 2020-01-13

**Authors:** Dongliang Ruan, Liming Qin, Rouxi Chen, Guojie Xu, Zhibo Su, Jianhua Cheng, Shilei Xie, Faliang Cheng, Frank Ko

**Affiliations:** 1 Guangdong Engineering and Technology Research Centre of Advanced and Nanomaterials, Dongguan University of Technology, Dongguan, 523808 China; 2 Dongguan Beyclean Environmental Protection Science and Technology Co. Ltd., Dongguan, 523690 China; 3 South China Institute of Collaborative Innovation, Dongguan, 523808 China; 4 Department of Materials Science and Engineering, Southern University of Science and Technology, Shenzhen, 518055 China; 5 State Key Laboratory of Precision Electronic Manufacturing Technology and Equipment; Guangdong Provincial Key Laboratory of Micro-nano Manufacturing Technology and Equipment, Guangdong University of Technology, Guangzhou, 510006 China; 6 Department of Material Engineering, University of British Colombia, Vancouver, V6T1W9 Canada

**Keywords:** Particulate matter (PM) pollution, Aerosol filtration, Electrospinning, Nanofiber membrane

## Abstract

Particulate matter is one of the main pollutants, causing hazy days, and it has been serious concern for public health worldwide, particularly in China recently. Quality of outdoor atmosphere with a pollutant emission of PM2.5 is hard to be controlled; but the quality of indoor air could be achieved by using fibrous membrane-based air-filtering devices. Herein, we introduce nanofiber membranes for both indoor and outdoor air protection by electrospun synthesized polyacrylonitrile:TiO_2_ and developed polyacrylonitrile-co-polyacrylate:TiO_2_ composite nanofiber membranes. In this study, we design both polyacrylonitrile:TiO_2_ and polyacrylonitrile-co-polyacrylate:TiO_2_ nanofiber membranes with controlling the nanofiber diameter and membrane thickness and enable strong particulate matter adhesion to increase the absorptive performance and by synthesizing the specific microstructure of different layers of nanofiber membranes. Our study shows that the developed polyacrylonitrile-co-polyacrylate:TiO_2_ nanofiber membrane achieves highly effective (99.95% removal of PM2.5) under extreme hazy air-quality conditions (PM2.5 mass concentration 1 mg/m^3^). Moreover, the experimental simulation of the test in 1 cm^3^ air storehouse shows that the polyacrylonitrile-co-polyacrylate:TiO_2_ nanofiber membrane (1 g/m^2^) has the excellent PM 2.5 removal efficiency of 99.99% in 30 min.

## Highlights

- Development of transparent PAN:TiO_2_ and PAN-co-PMA:TiO_2_ nanofiber membranes
- Synthesis and controlling of the properties of nanofiber membranes by electrospinning
- Strong PM adhesion and absorptive performance with the specific microstructure
- Nanofiber membrane shows excellent PM2.5 removal efficiency (99.99%) in 30 min

## Introduction

The particulate matter (PM) pollution issues are mainly caused by the high pollution manufacturing industry and are serious concerns worldwide, especially in China recently [1, 2]. Due to the severe environmental issues, people wear masks to filter pollute air outdoors in polluted weather conditions, and further equipment for air filtration becomes popular to clean indoor air quality in metropolises [3]. Right now, non-woven fibrous media have been used in different air filtration applications, from indoor air filter to personal protective equipment, such as N95 respirator. High-filtration efficiency or low-pressure drop is conducive to improve the quality of air filtration [4–7]. Non-woven microfibers with smaller diameter leads to not only greater filtration efficiency, but also larger pressure drop. For example, nanofiber-based air filters with a diameter smaller than 500 nm have high-filtration efficiency and low air permeability [8]. Therefore, the development of a high-performance nanofiber air filter membrane garners enormous interests from both research and applications worldwide, since nanofibers are rapidly becoming a feasible material alternative.

Among many approaches such as molecular technology, biological preparation, and spinning technique, electrospinning is a relatively simple and effective method, and also suitable and compatible with the preparation of nanofiber membranes [9–12]. Recently, nanofiber membranes have been successfully produced using different polymers by electrospinning for indoor air protection [13, 14]. Compared to other polymer materials, as PVA (polyvinyl alcohol), PS (polystyrene) and PVP (polyvinylpyrrolidone), the studies indicate that PAN (polyacrylonitrile) is a preferred material for particle filtration [15]. Moreover, some additional materials are easily coated on electrospun nanofibers, such as ZnO, TiO_2_, carbon nanotubes, silica, and silver. The artificial functional materials have been modified on different surfaces to increase the roughness and micro-nano structure [16, 17]. Among various coating materials, nanostructured TiO_2_ has received considerable interest, due to its remarkable UV-ray catalysis and shielding property [18–20]. The aim of the study is to develop electrospun nanofibers with rough surface, low-filtration pressure and resistance, which can actively capture PM2.5 based on the multi-stage structure of nanofiber membranes.

Therefore, we present an approach for the fabrication of polyacrylonitrile (PAN):TiO_2_ and developed polyacrylonitrile-co-polyacrylate (PAN-co-PMA):TiO_2_ nanofiber membrane by electrospinning (as shown in Suppl. Scheme 1.). The hierarchical PAN:TiO_2_ and particularly, PAN-co-PMA:TiO_2_ nanofiber membrane exhibited excellent filtration efficiency and good permeability, which is promising for air filter applications.

## Methods

### Materials

Polyacrylonitrile (PAN, MW: 100000) and polyacrylonitrile-co-polymethyl acrylate (PAN-co-PMA, MW: 150000) were purchased from Scientific Polymer; Polyvinylpyrrolidone (PVP, mw=55000) was purchased from Sigma; N,N-dimethyl formamide (DMF) was purchased from Anachemia; Nanometer titanium dioxide (TiO_2_, Anatase, D < 25 nm) was purchased from Aldrich. All raw materials were used as received without further purification.

### Electrospinning for Nanofiber Membrane

The PAN:TiO_2_ nanofiber membrane was fabricated by electrospinning. In the procedure, nanometer TiO_2_ and PVP (1:1, w/w) were added to DMF, and then PAN and PAN-co-PMA was added with final concentration of 10% (w/w). The mixture was heated and stirred to form a milk-white viscous solution for 24 h at 90°. The viscous solution was loaded into a plastic syringe equipped with an 18-gauge stainless steel needle. During electrospinning, the needle was supplied with a high positive electrostatic voltage. The ground collector was covered by PP nonwovens at a distance of 20 cm to the spinneret. The PAN:TiO_2_ and PAN-co-PMA:TiO_2_ nanofiber membranes were fabricated in a relative humidity of 45% at 25°. After electrospinning, the PAN:TiO_2_ and PAN-co-PMA:TiO_2_ nanofiber membranes were covered by another piece of nonwovens to protect the surface from damage. This composite membrane was dried in an oven for 3 h at 90°.

### Analysis

Scanning electron microscope (SEM) images were taken by a field emission SEM S3000N (Hitachi, Japan) and Transmission electron microscopy (TEM) images were taken by Hitachi H7600 (Japan). The crystal structure was characterized by X-ray diffraction (XRD) using a Rigaku X-ray diffractometer with graphite monochromatized Cu Kα irradiation (MultiFlex XRD, Japan). The diameter of nanofiber was measured using Image J software. The pore size of membranes was characterized by (Pore tester CFP-1100-AIP, MI). Fourier-transform infrared spectroscopy **(FTIR)** is from PerkinElmer (Frontier, PE, USA). Air permeability was measured using automatic air permeability meter (NingFang YG461E-111, China). The pressure drop and PM concentration were measured using PM Concentration 2.5 Tester (DustTrack 8520 TSI). PM particle number concentration was detected by laser particle counter (Purific Y09-301, China) and the removal efficiency was calculated by comparing the concentration before and after filtration. The photograms were captured by a digital camera (Nikon, D90).

## Results and Discussion

### Structure and Composition of Nanofiber Membrane

**Fig. 1:**
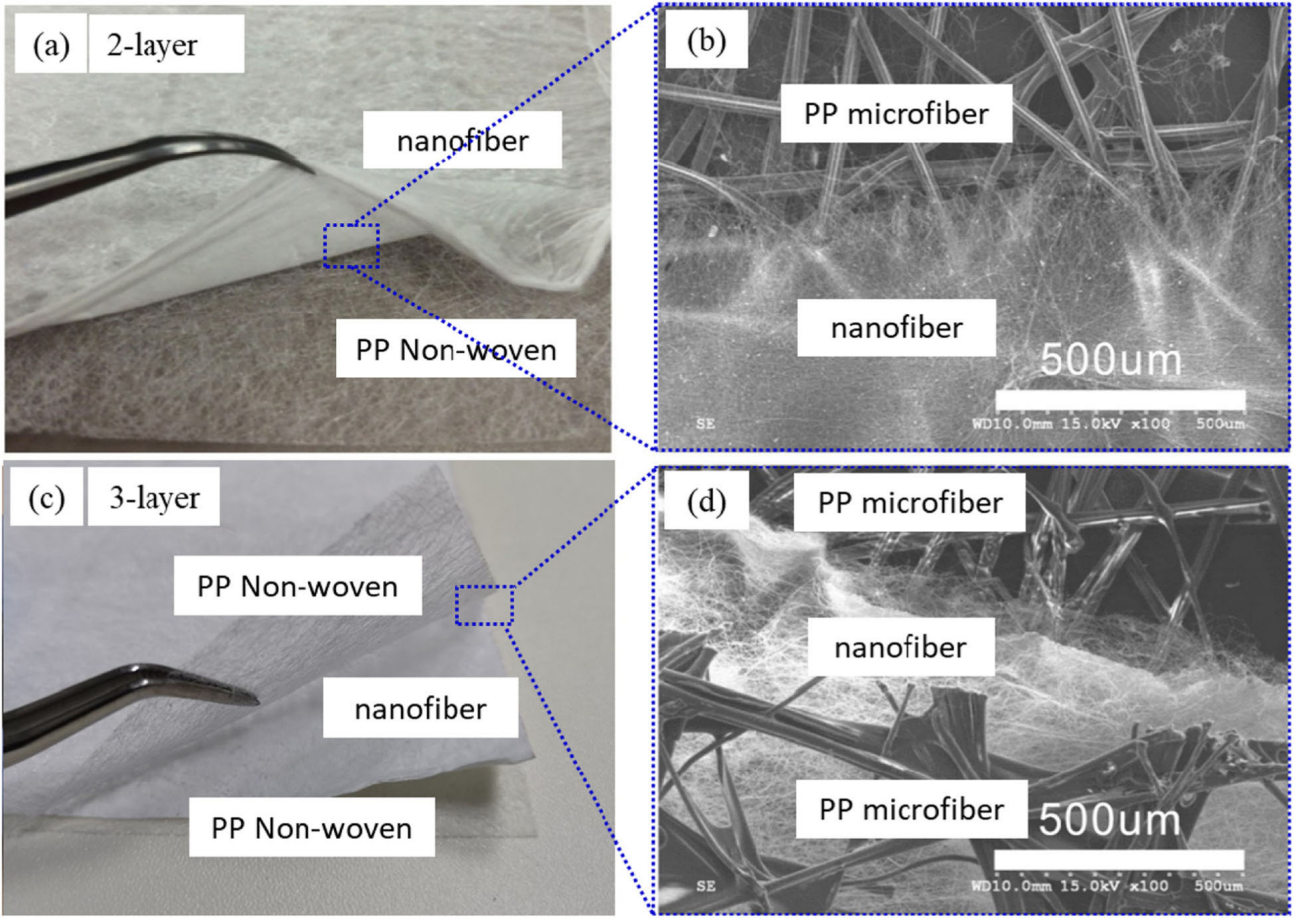
Morphology of PAN:TiO_2_ and PAN-co-PMA:TiO_2_ nanofiber membrane composited with PP non-woven air filter (layers): optical photograph of nanofiber membranes of 2-layer (**a**) and 3-layer (**c**), and their enlarged top-views (**c**, **d**), respectively

The typical nanofiber composite membranes of the optical images of 2 layers, 3 layers, and their SEM images were shown in Fig. 1a–d, respectively. The nanofiber membrane and the PP non-woven fabric support was layered, but the binding force was strong, because static electricity accumulates between the PP non-woven fabric and the nanofiber membrane during the electrospinning process. For example, we saw the layers of nanofiber and PP non-woven clearly in the 2-layer PAN:TiO_2_ nanofiber membrane (Fig. 1a), and top-view of the nanofiber membrane displayed PP microfiber and nanofibers structures obviously as shown in Fig. 1b. The structure of fabrication for a 3-layer was similar. We observed 3 layers’ structure (PP non-woven, nanofiber, and PP non-woven) and the first nanofiber layer was entangled with the non-woven fabric support in the SEM of the PAN:TiO_2_ nanofiber membrane, as shown in Fig. 1b, d.

**Fig. 2:**
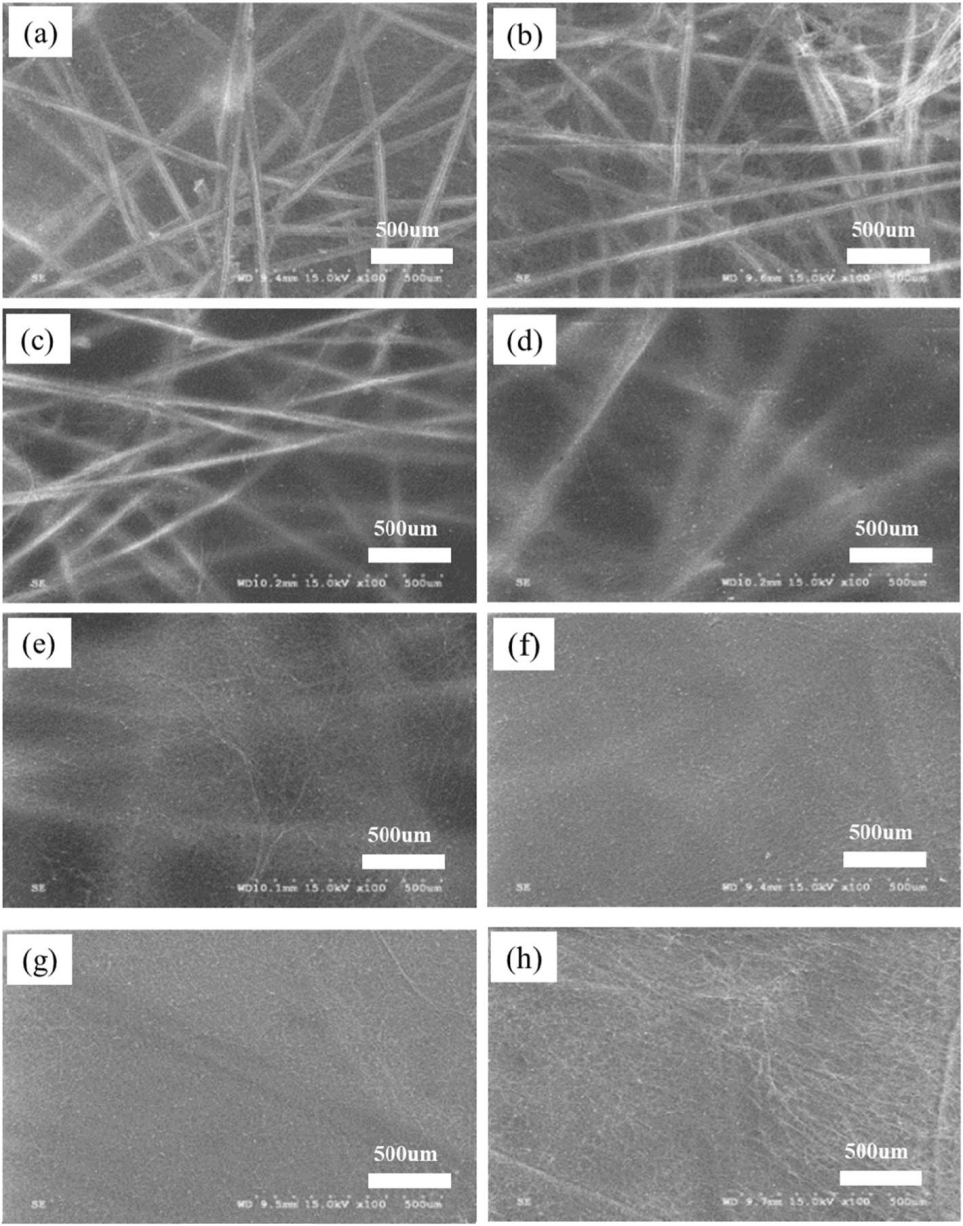
Morphology of PAN:TiO_2_ nanofiber membranes with different spinning times (different thicknesses): **a** 15 min, **b** 30 min, **c** 45 min, **d** 1 h, **e** 2 h, **f** 4 h, **g** 6 h and **h** 8 h

In order to synthesize the designed nanofiber membranes, we have developed and further optimized the approach by tuning the electrospinning parameters, such as spinning time, the receiving distance, temperature and humidity, voltage, traverse speed and rotation speed of the receiving roller. In the synthesizing process, we found that spinning time was controlling the thickness of nanofiber membranes, if we kept other electrospinning parameters unchanged. The shorter spinning time produced thinner nanofiber membranes. We produced a different thickness of nanofiber membranes by using different spinning time, as shown in Fig. 2. From the imagines of short spinning times as 15, 30, and 45 min, the skeleton of PP nonwoven was observed clearly in the nanofiber membrane (Fig. 2a–c). As the spinning time increasing to 1 and 2 h, the PP non-woven skeleton gradually became unclear and blurred, as shown in Fig. 2d, e, respectively. Finally, the visibility of the nonwoven fabric skeleton became hardly being observed, when the spinning time was as long as 4, 6, and 8 h (Fig. 2f–h).

In the SEM and TEM of PAN:TiO_2_ nanofiber membrane, the 3-layer one displayed the cross-sectional structure in the nanofiber membranes and nanofiber layer bonded to the non-woven fabric support (Additional file 1: Figure S1 in supporting data). The nanofibers have prominent TiO_2_ nanoparticles on the surface, which can be clearly observed in the TEM imagine (Additional file 1: Figure S1C). EDS, XRD, and FTIR identified that TiO_2_ nanoparticles were located on the surface and inside of the nanofibers in the anatase forms (Additional file 1: Figure S2–4 in supporting data).

**Fig. 3:**
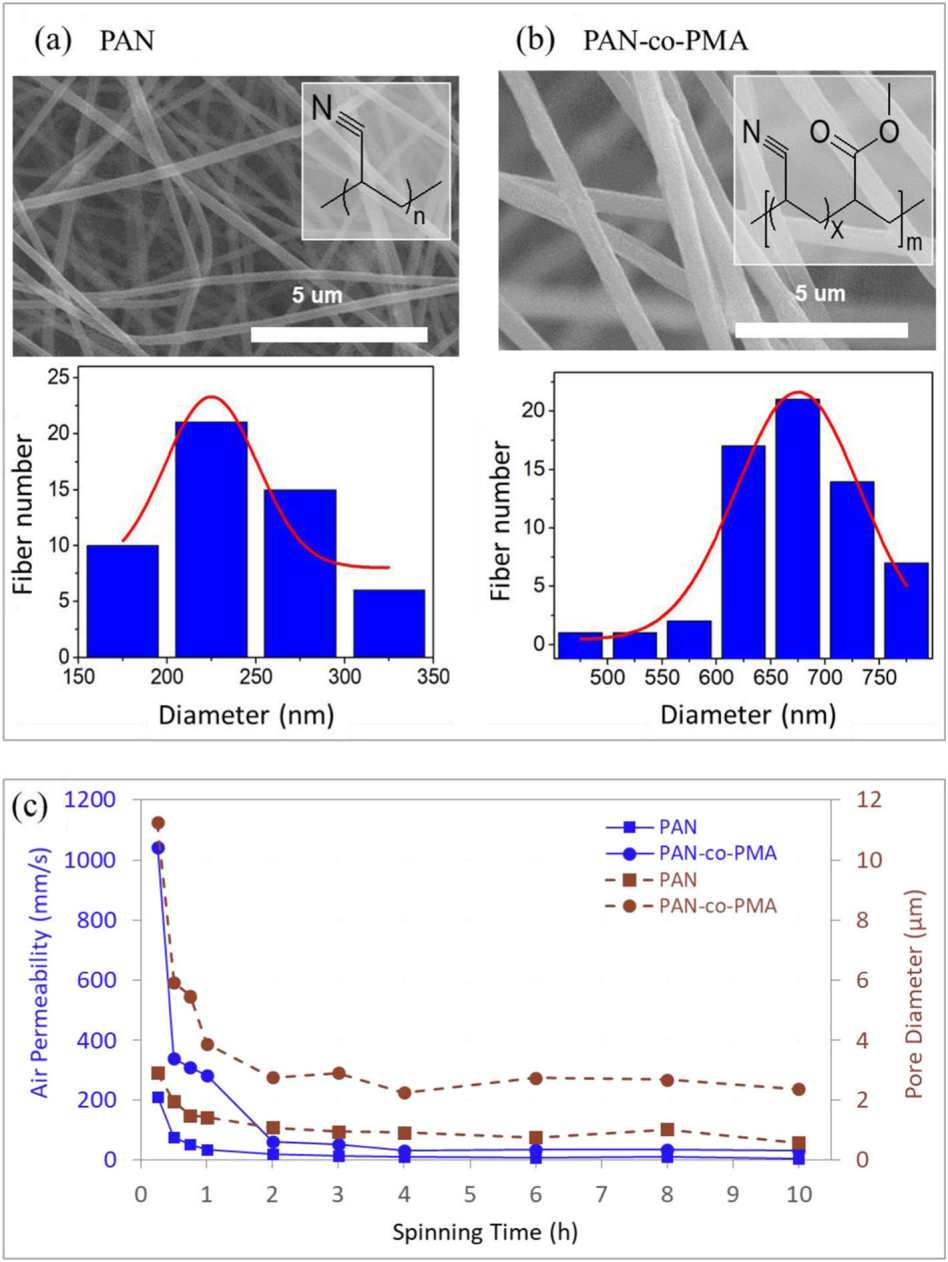
Diameter distribution of different PAN type (3% TiO_2_) nanofibers: (**a**) PAN:TiO_2_, (**b**) PAN-co-PMA:TiO_2_, and (**c**) average pore size and permeability of PAN:TiO_2_ and PAN-co-PMA:TiO_2_ nanofiber membranes

In PAN membranes, the fiber diameter ranged from 100 to 400 nm (average 237 nm) and the average molecular weight was around 100,000 Da. In PAN-Co-PMA membrane, the fiber diameter was 400~800 nm (average 678 nm) and an average molecular weight of 150,000. Because of the difference in molecular weight, it was clearly observed that the average and ranges diameters between the PAN:TiO_2_ and PAN-Co-MA:TiO_2_ nanofiber membranes are certainly different, as shown in Fig. 3a, b. The size of the fiber diameter influences the pore size and air permeability of the nanofiber membrane, in addition to the particle filtration efficiency and pressure drop of the nanofiber membrane, as shown in Fig. 3c. Due to the smaller fiber diameter, the pore size of PAN:TiO_2_ nanofiber membranes were smaller than PAN-co-PMA:TiO_2_ nanofiber membranes. Compared to the thickness of membrane, the nanofiber diameter had a larger influence on membrane pore size. Although thickness had a strong effect for the pore size of the nanofiber membrane (spinning time in 1 h), it only slightly changed the pore diameter, after the thickness reached a critical point (the spinning time longer than 2 h), as shown in Fig. 3c. It was similar to the air permeability of the nanofiber membrane, and the air permeability dropped with longer spinning time (membrane thicker), and membranes reached a plateau, when spinning time of 2 h. The air permeability of PAN:TiO_2_ nanofiber membranes was much lower than that of PAN-co-PMA:TiO_2_ when electrospun for 2–10 h. However, the variance of air permeability of PAN-co-PMA:TiO_2_ nanofiber membranes (32–35 mm/s) was higher than PAN:TiO_2_ nanofiber membranes (6–10 mm/s). It was probably due to the PAN:TiO_2_ nanofiber membrane (smaller diameter) deposit densely under similar spinning durations compared to the PAN-co-MA:TiO_2_ nanofibers. Therefore, the smaller nanofiber diameter and pore size of the nanofiber membrane experienced decreased flux, causing low air permeability Additional file 1: Figure S5.

### Applications for Particles Purification

**Fig. 4.**
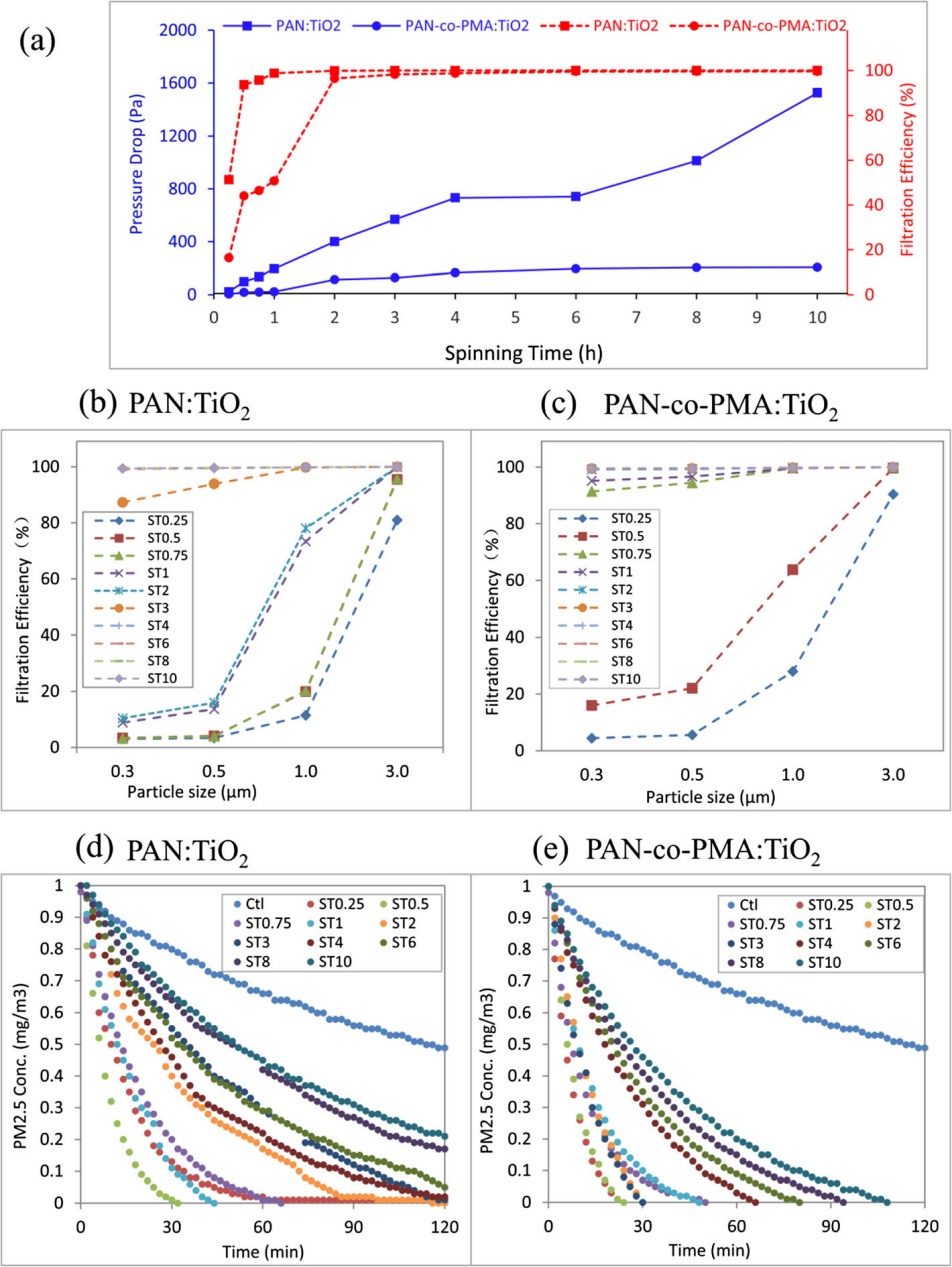
PAN:TiO2 and PAN-co-PMA:TiO2 nanofiber membranes’ filtration efficiency with (**a**) pressure drop of aerosols (**a**) and particle size (**b**, **c**); and the removal capability of (**d**) PAN:TiO2 and (**e**) PAN-co-PMA:TiO2 nanofiber membranein simulated polluted air test

The aerosol filtration efficiency and the pressure drop of PAN:TiO_2_ and PAN-co-PMA:TiO_2_ nanofiber membrane were studied. For both of nanofiber membrane, as the spinning time increased from 15 min to 2 h, the aerosol filtration efficiency increased sharply from as low as ~ 20 to 97% of and 50% for PAN-co-PMA:TiO_2_ and ~ 50 to 99% for PAN:TiO_2_, respectively (in Fig. 4a). The filtration efficiency of both nanofiber membranes was close to 100% if the spinning time was longer than 3 h. Meanwhile, the pressure drop increased with longer spinning time (thickness increasing). In the study, PAN:TiO_2_ nanofiber membrane continuously increased quickly to 600 Pa, when the spinning time was longer than 3 h, even reached 1000 Pa (spinning time longer than 8 h). However, the PAN-co-PMA:TiO_2_ nanofiber membrane increased much slow and kept the pressure drop around 200. Compared to the PAN-co-PMA:TiO_2_ nanofiber membrane, PAN:TiO_2_ membrane had smaller diameter and pore size and the membrane blocked the aerosol particles. At the same time, the smaller pore size caused the limited air permeability and higher pressure drop to maintain gas flow.

In the filtration efficiency study for different size particles, we generated simulated polluted air in hazy days by burning cigarettes and it contained CO, CO_2_, NO_2_, and volatile organic compounds, such as tar, nicotine, formaldehyde, and benzene. In the studied model system, we found that the thickness (spinning time) of nanofiber membrane had a strong effect of the filtration efficiency. For example, the filtration efficiency of PAN:TiO_2_ nanofiber membrane was higher than 90% if the spinning time was longer than 45 min, or close to 100%, if the spinning time was longer than 2 h) for the all tested particles at diameter from 0.3 to 3 μm, as shown in the Fig. 4b. Compared to PAN:TiO_2_ nanofiber membrane, the overall filtration efficiency of PAN-co-PMA:TiO_2_ nanofiber membrane was lower if the spinning time was shorter than 3 h. The filtration efficiency was also close to 100% for all the tested particles, if the spinning time was longer than 4 h in our study (Fig. 4c). The results of the filtration efficiency for both nanofiber membranes were similar to aerosol results. The large fiber diameter caused the big porosity between the fibers, increasing the possibility of particles passing through. The filtration efficiency on particulate matter reached a plateau, when the membrane thickness was to a certain level.

Further, we studied PM2.5 removal process of PAN:TiO_2_ and PAN-co-PMA:TiO_2_ nanofiber membranes for 2 h, and the field tests were in a 1-m^3^ chamber of real polluted air environment. The model system of the air chamber was designed (shown in Additional file 1: Figure S6) and the initial PM2.5 concentration was 1 mg/m^3^. We used the circular nanofiber composite membranes for PM2.5 filtration and the PM2.5 particles in the air chamber were recorded every minute in total 120 min. The result of two nanofiber membranes was shown in Fig. 4d, e. PAN-co-PMA:TiO_2_ nanofiber membranes removed all PM2.5 in 120 min, and thinner (spinning time ≤ 2 h) completely reduced PM2.5 in 50 min, and membranes with electrospinning time of 0.25 h and 0.5 h even filtered all PM2.5 in about 20 min. PAN:TiO_2_ nanofiber membranes had better removal of PM2.5 in the tests, and the membranes (electrospinning time > 4 h) could not reduce the PM2.5 in 2 h, as shown in Fig. 4e. Generally, PAN-co-PMA:TiO_2_ nanofiber membrane had higher removal of PM2.5 than that of PAN:TiO_2_ nanofiber membrane.

## Conclusion

In summary, we synthesized the PAN:TiO_2_ and PAN-co-PMA:TiO_2_ nanofiber membranes by using electrospinning and the properties of nanofiber membranes, as air permeability, aerosol test, and PM trapping were systematically evaluated. The microfiber non-woven, the nanofiber membrane, and the non-woven fabric bracket were well composited into a multi-layer structure by electrostatic force for two types of nanofiber membranes. The bonding structure of PAN-co-PMA:TiO_2_ nanofiber membrane displayed excellent air permeability (284–339 mm/s) and removal of PM2.5. Moreover, the developed nanofiber membranes were cost-effective and practical PM2.5, which would be applicable as a commercial air purifier filter to prevent PMs in the future.

## Supplementary information

**Additional file 1: Figure S1.** (a) SEM of cross-sectional PAN@TiO_2_ nanofiber membrane (b) SEM at 10 μm and (c) TEM imagine at 500 nm of PAN@TiO_2_ nanofiber membrane. (TiO_2_ content of 3%). **Figure S2.** EDS image of PAN@TiO_2_ nanofiber membrane. **Figure S3.** EDS image of C Kα1 (a) and Ti Kα1 (b). **Figure S4.** XRD of PAN-TiO_2_ nanofiber membrane. **Figure S5.** FTIR of PAN:TiO2 and PAN-co-PMA:TiO2 NFM(Nanofiber Membrane). **Figure S6.** Simulated polluted air test device. **Figure S7.** SEM of PAN nanofibers with(a) and without (b) the TiO_2_, PM2.5 filtration efficiency of PAN nanofibers &PAN:TiO2 nanofibers in Simulated polluted air test device (120min).

## Data Availability

Please find the availability of data in supporting data.

## Abbreviations

DMF: N,N-dimethyl formamide
FTIR: Fourier-transform infrared spectroscopy
PAN: Polyacrylonitrile
PAN-co-PMA: Polyacrylonitrile-co-polyacrylate
PM: Particulate matter
PS: Polystyrene
PVP: Polyvinylpyrrolidone
SEM: Scanning electron microscope
TEM: Transmission electron microscopy
VA: Polyvinyl alcohol
XRD: X-ray diffraction
