# Supplementary material for: Transparent PAN:TiO2 and PAN-co-PMA:TiO2 Nanofiber Composite Membranes with High Efficiency in Particulate Matter Pollutants Filtration
Source: Nanoscale Res Lett. 2020 Jan 13;15:7. doi: 10.1186/s11671-019-3225-2 (PMC6957606; doi:10.1186/s11671-019-3225-2)
Supplement: Supplementary file 1 — Additional file 1: Figure S1. (a) SEM of cross-sectional PAN@TiO2 nanofiber membrane (b) SEM at 10 μm and (c) TEM imagine at 500 nm of PAN@TiO2 nanofiber membrane. (TiO2 content of 3%). Figure S2. EDS image of PAN@TiO2 nanofiber membrane. Figure S3. EDS image of C Kα1 (a) and Ti Kα1 (b). Figure S4. XRD of PAN-TiO2 nanofiber membrane. Figure S5. FTIR of PAN:TiO2 and PAN-co-PMA:TiO2 NFM(Nanofiber Membrane). Figure S6. Simulated polluted air test device. Figure S7. SEM of PAN nanofibers with(a) and without (b) the TiO2, PM2.5 filtration efficiency of PAN nanofibers &PAN:TiO2 nanofibers in Simulated polluted air test device (120min). [file 11671_2019_3225_MOESM1_ESM.docx]

Additional file 1: Figure S1.


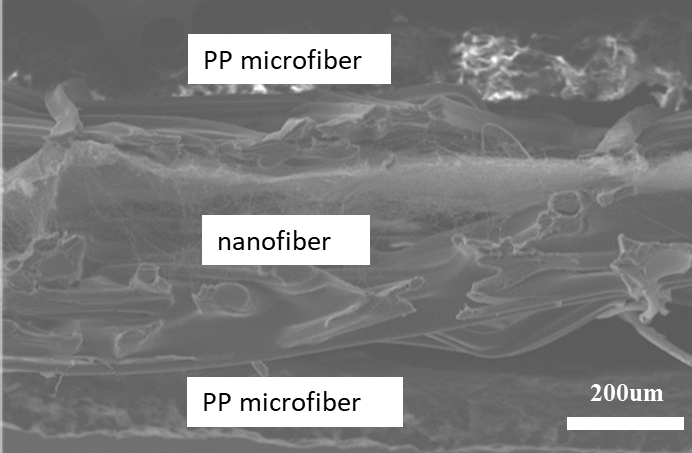


(a)


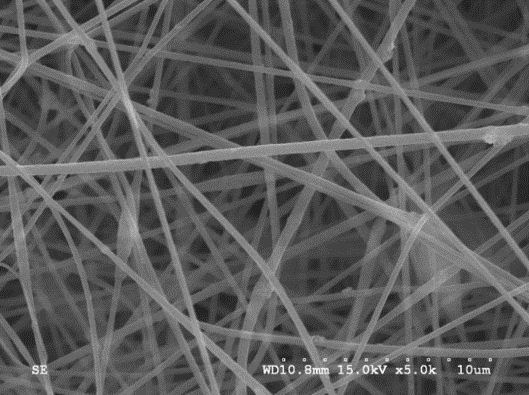


**10um**

(b)

(c)


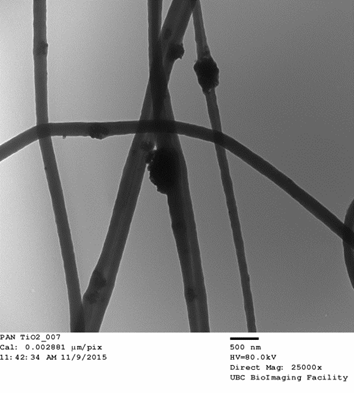


**500nm**

Additional file 1: Figure S1. (a) SEM of cross-sectional PAN@TiO_2_ nanofiber membrane (b) SEM at 10 µm and (c) TEM imagine at 500 nm of PAN@TiO_2_ nanofiber membrane. (TiO_2_ content of 3%).

Additional file 1: Figure S2.


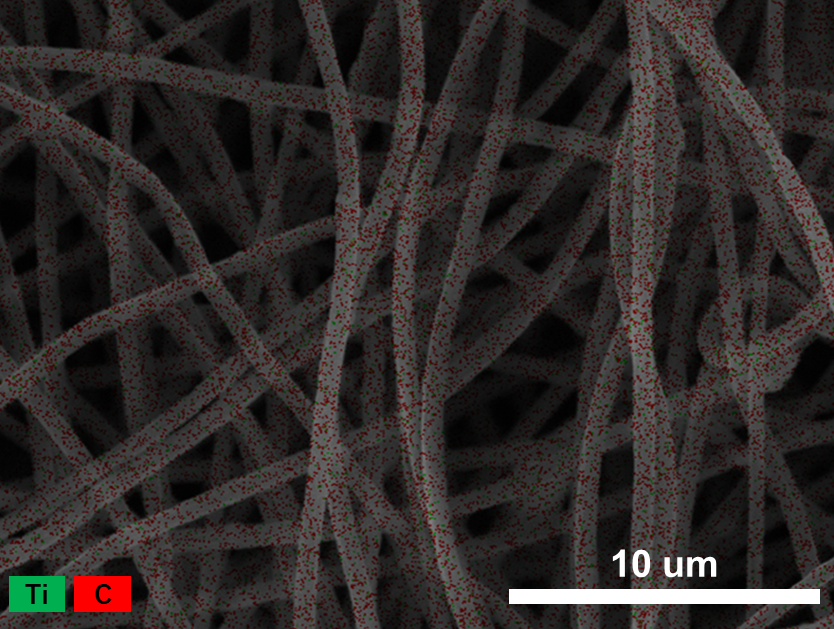


Additional file 1: Figure S2. EDS image of PAN@TiO_2_ nanofiber membrane

Additional file 1: Figure S3.


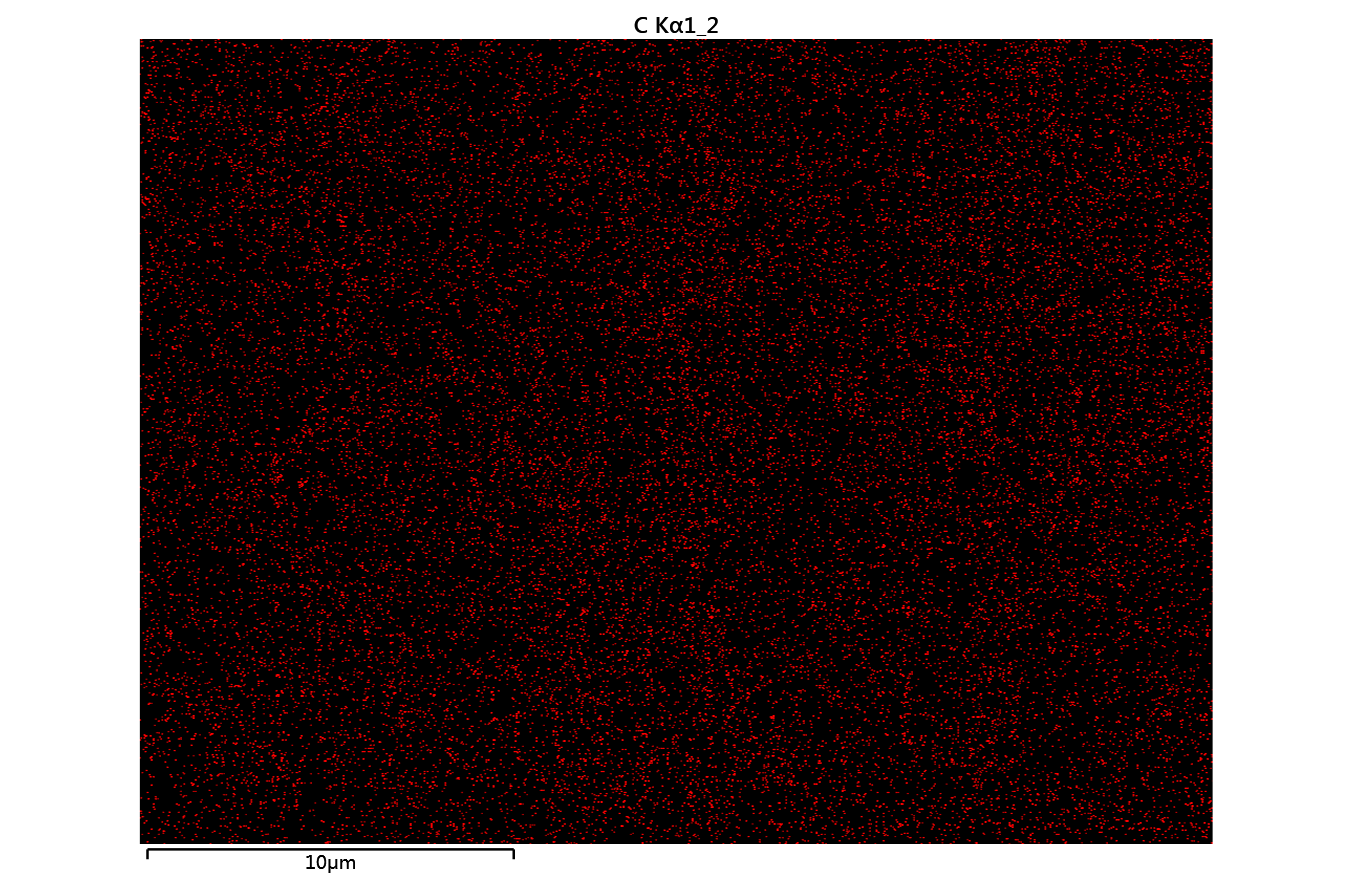


(a)


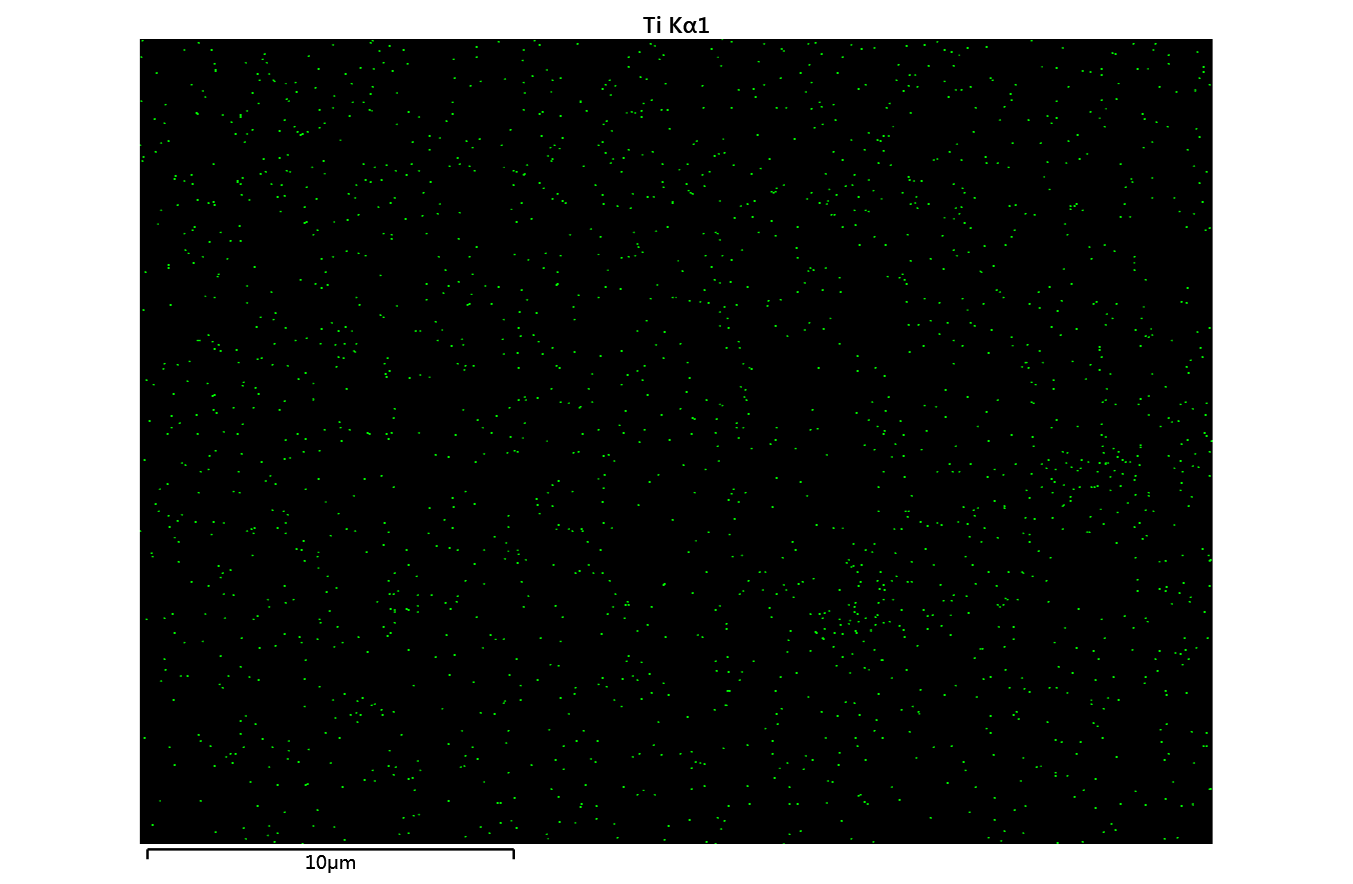


(b)

Additional file 1: Figure S3. EDS image of C K$\alpha$1 (a) and Ti Kα1 (b).

Additional file 1: Figure S4.


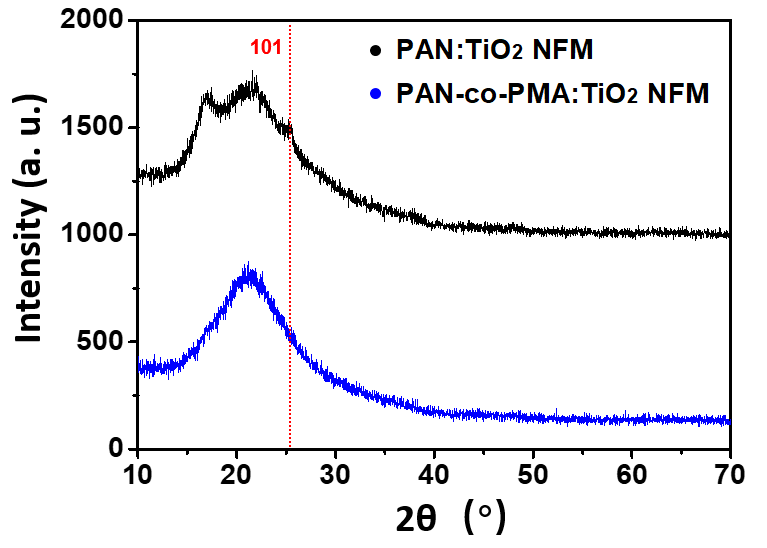


Additional file 1: Figure S4. XRD of PAN-TiO_2_ nanofiber membrane.

Additional file 1: Figure S5.


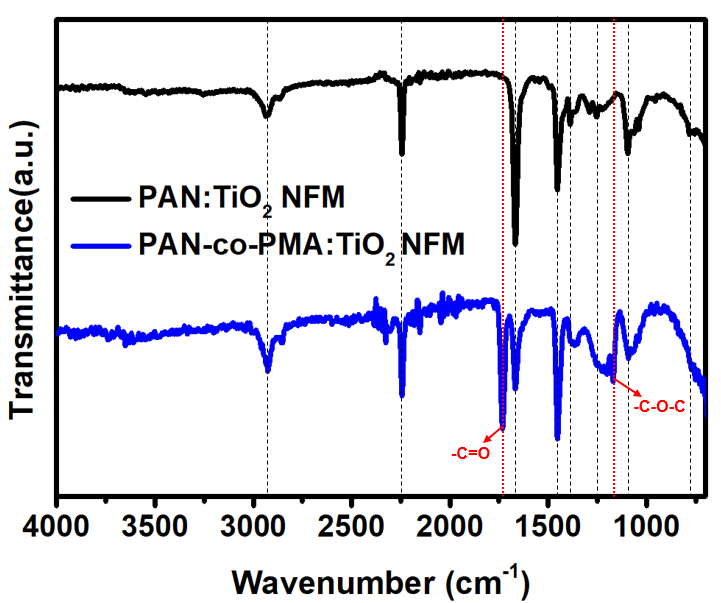


Additional file 1: Figure S5. FTIR of PAN**:**TiO2 and PAN-co-PMA**:**TiO2 NFM（Nanofiber Membrane）.

Additional file 1: Figure S6.


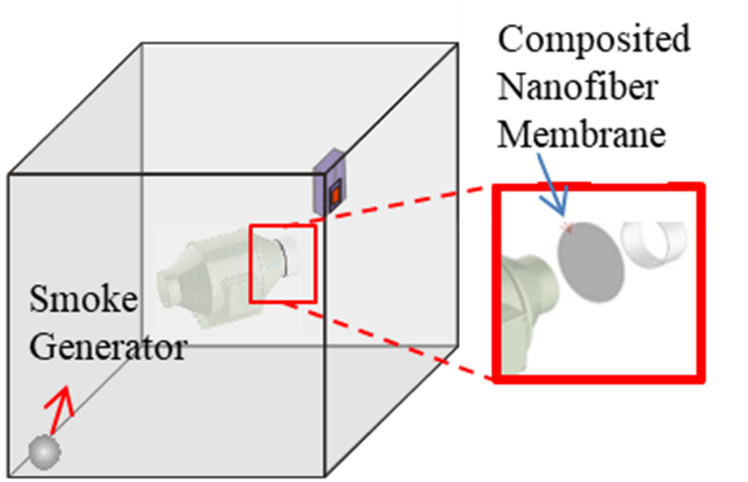


Additional file 1: Figure S6. Simulated polluted air test device.

Additional file 1: Figure S7.


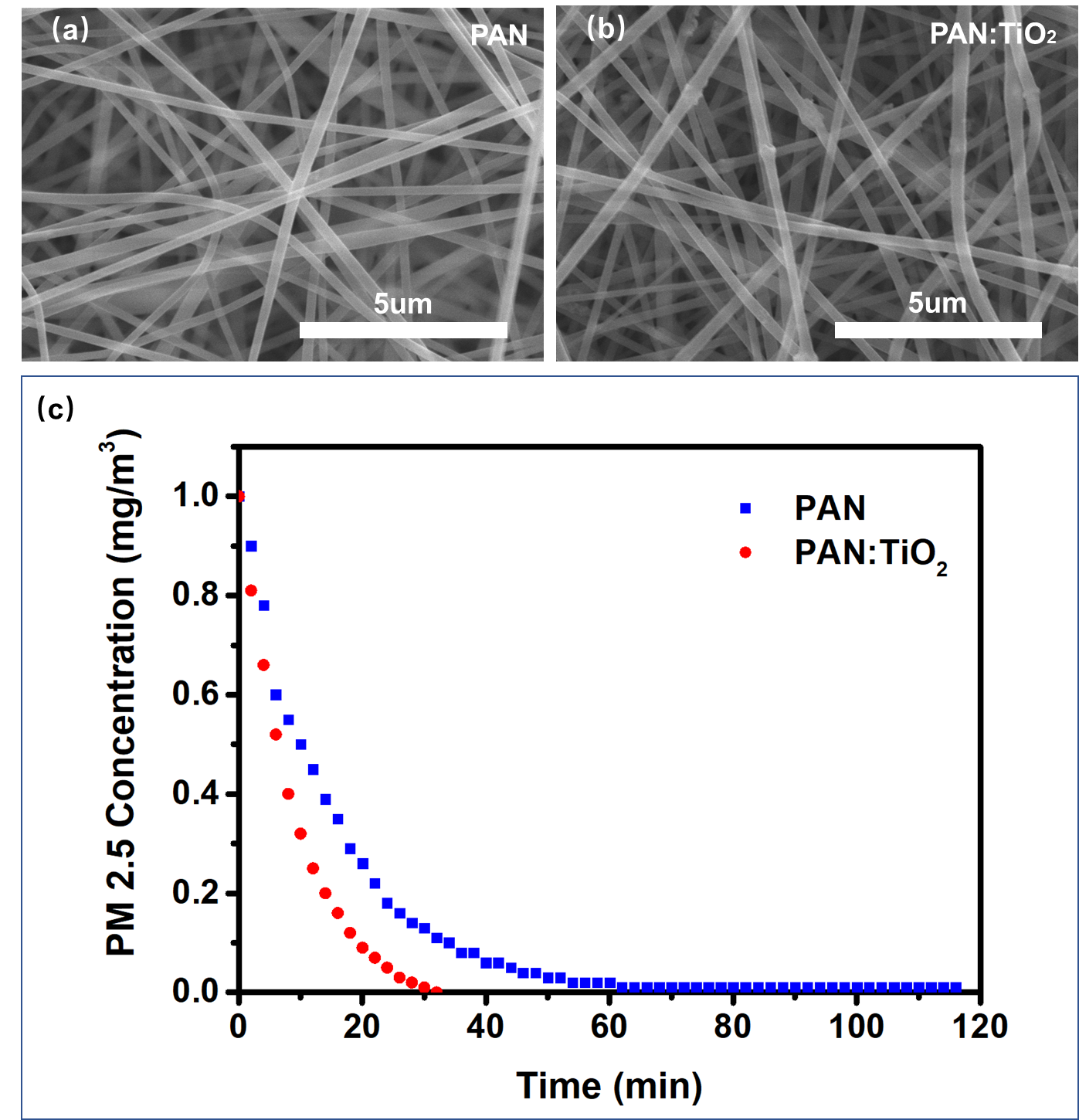


Additional file 1: Figure S7. SEM of PAN nanofibers with(a) and without(b) the TiO_2_, PM2.5 filtration efficiency of PAN nanofibers &PAN：TiO2 nanofibers in Simulated polluted air test device (120min)
